# *IGLL5* controlled by super-enhancer affects cell survival and *MYC* expression in mature B-cell lymphoma

**DOI:** 10.1016/j.lrr.2024.100451

**Published:** 2024-02-22

**Authors:** Hiroki Hosoi, Shotaro Tabata, Hideki Kosako, Yoshikazu Hori, Tadashi Okamura, Yusuke Yamashita, Kota Fujimoto, Daiki Kajioka, Kentaro Suzuki, Motomi Osato, Gen Yamada, Takashi Sonoki

**Affiliations:** a Department of Hematology/Oncology, Wakayama Medical University, Wakayama, Japan; b Department of Developmental Genetics, Institute of Advanced Medicine, Wakayama Medical University, Wakayama, Japan; c Faculty of Life and Environmental Sciences, University of Yamanashi, Takeda 4-4-37, Kofu City, Yamanashi, Japan; d International Research Center for Medical Sciences, Kumamoto University, Kumamoto, Japan

**Keywords:** Mature b-cell lymphoma, Immunoglobulin lambda like polypeptide 5 (igll5), Super-enhancer, SiRNA

## Abstract

*IGLL5* is shown to be located near super-enhancer (SE) in B-cell tumors, and this gene is frequently mutated and a target of translocation in B-cell tumors. These results suggest roles of the *IGLL5* in tumorigenesis; however, its functional properties have been unclear. We found that two mature B-cell lymphoma cell lines expressed *IGLL5* mRNA with *Cλ1* segment. JQ1 treatment resulted in down-expression of *IGLL5*, indicating that *IGLL5* is controlled by SE. *IGLL5* knockdown induced cell death with down-expression of *MYC*. Our results suggested that *IGLL5* might have a role in survival of mature B-cell tumors and involvement in *MYC* expression. (100 words)

## Highlights

• Immunoglobulin light like polypeptides 5 gene (*IGLL5*) is expressed in mature-B-cell lymphoma lacking IGλ protein.
• *IGLL5* expression is controlled by super-enhancer, predicted by *in-silico* analyses.
• Decreased expression of *IGLL5* results in apoptosis with down-expression of *MYC*.

## 1 Introduction

Mature B-cell lymphoma is the most common among hematopoietic tumors. Accumulated evidence indicates that acquired genetic alterations are required for the disease development, and novel findings continue to identify molecular pathogenesis of this disease. In the past decade, genome-wide chromatin immunoprecipitated sequencing (ChIP-seq) has revealed that large active enhancers, called super-enhancers (SE), are scattered in the genome, and that tumor cell-specific SEs have been identified [1]. Most of the genes controlled by SE have been shown to play important roles in tumorous cells as well as in normal cells [1]. A high-ranked SE is predicted at the proximity of immunoglobulin lambda like polypeptide 5 gene (*IGLL5*) in diffuse large B-cell lymphoma or multiple myeloma [2,3]. *IGLL5* might therefore have some function in the tumorigenesis of B-cell malignancies.

*IGLL5* consists of three exons. The first exon is unique for *IGLL5* and located at ∼6 kb upstream of the immunoglobulin lambda (*IGλ*) joining region 1 segment (*Jλ1*) [4,5]. The second and third exons are *Jλ1* and *IGλ* constant region 1 segments (*Cλ1*), respectively. *IGLL5* mRNA therefore contains *Jλ1* and *Cλ1* nucleotide alignment. Authentic *IGλ* requires somatic rearrangements to express as mRNA encoding IGλ protein; but, *IGLL5* does not [4,5]. Two transcriptional isoforms of *IGLL5* have been identified, the isoforms 1 and 2 encode 214 and 139 amino-acids protein, respectively [6]. Both proteins belong to the immunoglobulin superfamily. Although many immunoglobulin superfamily proteins play significant roles in immunity, cell adhesion, and signaling [7], the function of IGLL5 has not been well explored.

*IGLL5* has recently been reported as one of the most frequently mutated genes in mature B-cell lymphoma and multiple myeloma [8,9,10]. The frequently-encountered mutated genes seen in B-cell tumors, such as *BCL2* or *EZH2*, have some functions in B-cell malignancies. In addition, *IGLL5* has also been shown to be a target of chromosome translocation in mature-B-cell lymphoma or multiple myeloma [10,11]. Generally, a target gene of chromosome translocation in hematopoietic tumors plays important roles in the tumorigenesis. These observations on *IGLL5*, including the proximity to SE, the genetic mutation, and the target gene of translocation, suggest that *IGLL5* might have some role in B-cell tumorigenesis. However, the biological role of the *IGLL5* in B-cell neoplasms remains unclear.

We found that two mature B-cell lymphoma cell lines expressed *IGLL5*. Using these cell lines, we attempted to analyze the biological role of *IGLL5* in mature B-cell lymphoma using small interfering RNA (siRNA) methods.

## 2 Materials and methods

### 2.1 Cell lines

Four mature B-cell lymphoma cell lines (MD901, FL218, WILL1 and WILL3), one myeloma cell line (RPMI8226) and one myeloid cell line (K562) were used in this study. MD901 and FL218 are positive for cell surface IGκ and IGλ protein, respectively. WILL1 produces IGκ protein, while WILL3 lacks surface IGs and produces no IG protein. RPMI8226 secretes IGG/IGλ protein. The characteristics of the four mature B-cell lines are summarized in the supplemental table.

### 2.2 Reagents and transfection

A bromodomain and extra terminal domain inhibitor, JQ1, was used as an SE inhibitor [2,3]. The subjected cells were incubated in culture medium containing JQ1 at final concentration 0.75 μM for 14 h or 24 h. To knockdown *IGLL5* mRNA, we used a previously reported *Cλ* siRNA pool, si[*IGLC*_CR_] [12]. Negative control siRNA (sense, 5′-UUCUCCGAA CGUGUCACGU-3′ and anti-sense, 5′-ACGUGACACGUUCGGAGAA-3′), designated as si[*Cont*], was purchased from elsewhere (Fasmac, Saitama, Japan). All siRNAs were transfected into cells by electroporation (Nepa-gene, Toyama, Japan) according to the manufacturer's protocol. Briefly, cells (5∼6 × 10^6^) at log-phase proliferation were collected by centrifugation at 160 g for 1 min at 4 °C. The cells were then washed with 20 ml of Opti-MEM medium (Life Technologies Companies, Grand Island, NY) and centrifuged at 160 g for 1 min at 4 °C. Finally, 1 × 10^6^ cells were suspended in 100 μl of Opti-MEM medium containing 50 pmol of siRNA, and then electroporated. The cells were then suspended in 2 ml of pre-warmed culture medium, and maintained in a well of the 24-well cell culture plate (IWAKI, Shizuoka, Japan).

### 2.3 Northern blot analysis

Total RNA was extracted using TRIzol Reagent (Invitrogen, Carlsbad, CA). One μg of total RNA except RPMI8226 was subjected to the analysis. For RPMI8226, 0.5 μg of total RNA was loaded. Northern blot analyses were performed using the DIG Northern Starter Kit according to the manufacturer's protocol. (Roche Diagnostics, Mannheim, Germany). Chemiluminescence signals were detected on X-ray films. A 0.7 kb of *IGλ3* constant (*Cλ3*) and a 2.2 kb of *MYC* cDNA fragments were obtained from the pC-lambda 3 and pSPT-myc cDNA, respectively (both were purchased from JCRB, Ibaraki, Japan). A 464 bp of DNA fragment encompassing *IGLL5* exon 1 was amplified by PCR using IGLL5F1 (5′-GTAGATGCCCCTCTGGGAGA-3′) and IGLL5R1 (5′- ACCTGGGGTCTGCTCTCTGG-3′) primers. These three DNA fragments were subcloned to appropriate plasmids that have Sp6 or T7 promoter at the cloning site for reading by RNA polymerase. Digoxigenin (DIG)- labelled RNA probes were synthesized using the DIG RNA Labeling Kit (SP6/T7) (Roche Diagnostics, Mannheim, Germany). The DIG-labelled RNAs derived from the respective DNA fragments were designated as Cλ, MYC and IGLL5 probes corresponding to the parental plasmids. The Cλ probe hybridizes to all *Cλ* segments because of its high homology.

### 2.4 5′ rapid amplification of cDNA end (5′RACE) and reverse transcriptase PCR (RT-PCR)

5′RACE was performed using the SMARTer RACE 5′/3′ Kit (TaKaRa Bio, Otsu, Japan) according to the manufacturer's protocols. Gene specific reverse primers for the first and the second rounds PCR were 5′-CTCCACGGTGCTCCCTTCATGCGTGACC-3′ and 5′-GTTTGGAGGGT(G/T)TGGT(G/C)GTC-3′, respectively. A ∼660 bp of 5′RACE product was subcloned into pGEM/T-easy vector (Promega, Madison, WI) and nucleotide alignments were determined. The nucleotide alignments were analyzed using the BLAST algorithm (https://blast.ncbi.nlm.nih.gov/). To detect *IGLL5* transcript, RT-PCR was performed. The open reading frame sequence of *IGLL5* was amplified using IGLL5F2 (5′-CCAATGGACTGGGGTGTACT-3′) and IGLL5R3 (5′-GAGAAGGGCTGGATGACTTG-3′) primers. We used a high-fidelity thermostable DNA polymerase (PrimeSTAR, TaKaRa Bio, Otsu, Japan). The PCR products of WILL1 and WILL3 were purified and then directly sequenced.

### 2.5 Apoptosis assay

The cells were analyzed 24 h after electroporation The subjected cells were stained with annexin-V and propidium iodine using the Annexin-V-FLUOS staining kit according to the manufacturer's protocol (Roche Diagnostics, Mannheim, Germany). The cells were then analyzed by flow cytometry (FACS, Beckton-Dickinson, Franklin Lakes, NJ). FACS data were analyzed by FlowJo software (Beckton-Dickinson, Franklin Lakes, NJ). Dead cells were counted as the sum of early apoptotic, late apoptotic and necrotic cells. Four independent analyses were performed, and statistical significance was examined by Student's *t*-test method.

## 3 Results

### 3.1 *IGLL5* mRNA expressed in mature B-cell lines lacking IGλ production

**Fig. 1:**
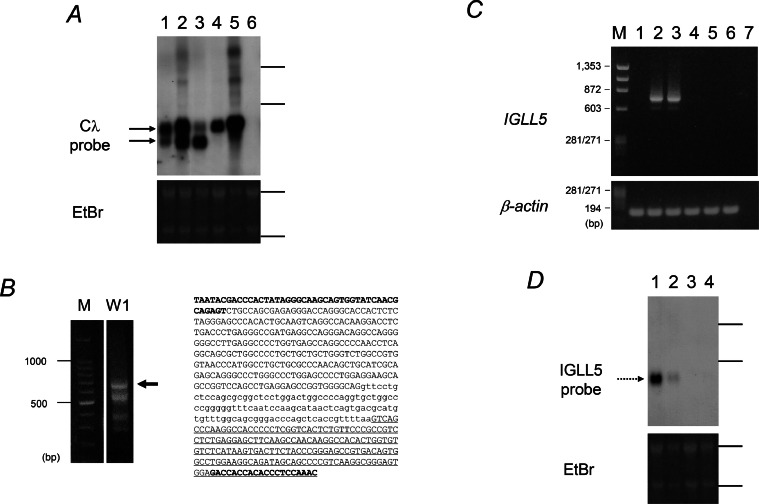
Detection of *IGLL5* mRNA in mature B-cell lymphoma lines. *A.* Northern blot analysis using the Cλ probe. Lane 1: MD901, lane 2: WILL1, lane 3: WILL3, lane 4: FL218, lane 5: RPMI8226, lane 6: K562. Ethidium bromide (EtBr) staining gel served as a loading control. Arrows indicate *Cλ* containing transcripts seen in IGλ negative cell lines. Horizontal bars represent position of 28S and 18S ribosomal RNAs. *B.* Results of 5′RACE. M: DNA size marker (100 bp DNA Ladder, TaKaRa Bio, Otsu, Japan); W1: 5′RACE products derived from WILL1 cDNA. The ∼660 bp of product (arrow) was subcloned and sequenced. The nucleotide alignments are shown on the right. Capital, small, and underlined letters indicate *IGLL5* exon 1, exon 2 (=*Jλ1*), and exon 3 (=*Cλ1*), respectively. Bald letters represent primers. The 5′RACE product showed 82.8 % of homology to *IGLL5. C.* Results of RT-PCR using the IGLL5F set on exon 1 and IGLL5R3 set on exon 3. M: DNA size marker (ϕ174/HaeIII digests), lane 1: MD901, lane 2: WILL1, lane 3: WILL3, lane 4: FL218, lane 5: RPMI8226, lane 6: K562, lane 7: water. β-actin provides an internal control. The PCR products obtained from WILL1 and WILL3 were purified and directly sequenced (Figure S1). *D.* Northern blot analysis using the IGLL5 probe. Lane 1: WILL1, lane 2: WILL3, lane 3: FL218, lane 4: RPMI8226. The dot arrow indicates *IGLL5* mRNA. EtBr staining gel served as a loading control.

We detected two transcripts containing *Cλ* segment in three IGλ negative cell lines (Fig. 1A). Owing to lack of IGλ protein synthesis, we thought that the transcripts do not encode IGλ protein. To determine the origin of the transcripts, we performed 5′RACE. The 5′RACE using WILL1 cDNA yielded multiple PCR products. The largest ∼660 bp fragment (Fig. 1B) was cloned and sequenced. The nucleotide alignments (Fig. 1B) showed homology to the *IGLL5* transcript by the BLAST search program. RT-PCR was then performed using IGLL5F2 and IGLL5R3 primers to validate *IGLL5* expression. The RT-PCR detected *IGLL5* transcripts in WILL1 as well as WILL3 (Fig. 1C). Direct sequencing of the PCR products revealed that both *IGLL5* mRNAs are identical to *IGLL5* transcript isoform 2 lacking the second exon (*Jλ1*) (Figure S1). Although 5′RACE detected the *IGLL5* transcript containing exon 2 in WILL1, (Fig. 1B), the main *IGLL5* transcript is considered to be isoform 2. The *IGLL5* mRNA is predicted to produce 139 amino-acids protein. Northern analysis confirmed that both of the cell lines express *IGLL5* mRNA (Fig. 1D).

### 3.2 Down-expression of IGLL5 mRNA by JQ1 and Cλ siRNA

**Fig. 2:**
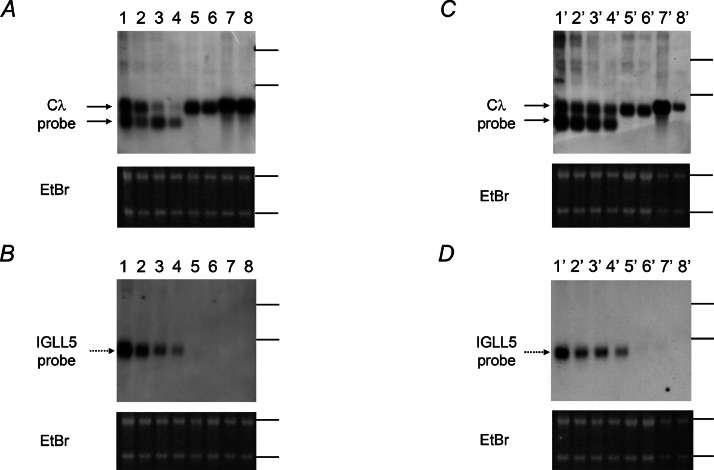
Northern blot analyses of *IGLL5* mRNA expression using the Cλ and IGLL5 probes after JQ1 treatment or Cλ siRNA transfection. *A, B*. Decreased signals of Cλ (A) and IGLL5 (B) probes after JQ1 treatment were observed. JQ1 was added in RPMI1680/10 %FBS medium at final concentration 0.75 μM. The same volume of DMSO was added as a control. The subject cells were collected after 14 h culture. Lanes 1 and 2: WILL1, lanes 3 and 4: WILL3, lanes 5 and 6: FL218, lanes 7 and 8: RPMI8226. Odd and even numbers represent DMSO and JQ1 treatment, respectively. EtBr staining gel served as a loading control. *C, D*. Decreased signals of the Cλ (C) and IGLL5 (D) probes after si[*IGLC*_CR_] transfection were observed. 50 pmol of si[*Cont*] or si[*IGLC*_CR_] were transfected by electroporation. The subject cells were cultured for 14 h after transfection. Lanes 1′ and 2′: WILL1, lanes 3′ and 4′: WILL3, lanes 5′ and 6′: FL218, lanes 7′ and 8′: RPMI8226. Odd and even numbers represent si[*Cont*] and si[*IGLC*_CR_] treatment, respectively. EtBr staining gel served as a loading control.

A high-ranked SE was predicted in previous reports near the *IGLL5* in diffuse large B-cell lymphoma and myeloma cells [2,3]. We also confirmed a large active Histone H3 at lysine 27 acetylation (H3K27Ac) region*,* suggesting SE, near *IGLL5* in mature B-cell lines using a published data-base, ChIP-Atlas (https://chip-atlas.org/) (Figure S2). This *in silico* data suggests that *IGLL5* expression is controlled by SE. To confirm regulation of *IGLL5* by SE, we examined expression levels after JQ1 treatment by Northern blot analyses using the Cλ and IGLL5 probes recognizing the third and first exons. The *IGLL5* mRNA expression in WILL1 and WILL3 was more sensitive to JQ1 treatment than the *Cλ* mRNA expression encoding IGλ protein in FL218 or RPMI8226 (Fig. 2A and B).

Next, we challenged knockdown of the *IGLL5* mRNA using a previously reported siRNA targeted *Cλ* region, si[*IGLC*_CR_], because the *Cλ1* is the third exon of *IGLL5*. The si[*Cont*] and si[*IGLC*_CR_] were introduced into cells by electroporation. Although the efficacy differed in each cell line, the signals derived from the Cλ probe decreased at 14 h after si[*IGLC*_CR_] transfection (Fig. 2C). RPMI8226 showed the most significant reduction of signals among the subjected cells. Signals derived from the IGLL5 probe were also decreased after transfection in WILL1 and WILL3 cells (Fig. 2D) . This result suggested that si[*IGLC_CR_*] can decrease *IGLL5* mRNA.

### 3.3 *IGLL5* knockdown induces apoptosis along with decreased expression of *MYC*

**Fig. 3:**
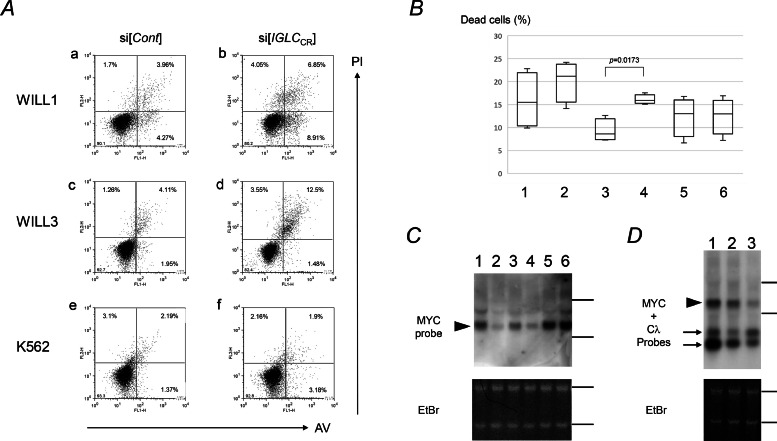
Apoptosis and *MYC* down-expression after si[*IGLC*_CR_] transfection. *A.* Apoptosis assay using annexin-V (AV) and propidium iodine staining (PI). The subject cells were transfected to si[*Cont*] or si[*IGLC*_CR_] by electroporation. The subject cells were cultured in RPMI1620/10 %FBS for 24 h after electroporation, then, apoptosis assays were performed. Lower right (LR), upper right (UR) and upper left (UL) quadrant represent early apoptotic cells, late apoptotic, and necrotic cells, respectively. Dead cells were counted as a sum of cells in LR, UR and UL quadrants. *B.* Increase in dead cells after si[*IGLC*_CR_] transfection. WILL3 showed statistically significant increase of dead cells after si[*IGLC*_CR_] transfection. Lanes 1 and 2: WILL1, lanes 3 and 4: WILL3, lanes 5 and 6: K562. Odd and even numbers represent si[*Cont*] and si[*IGLC*_CR_] transfection. *C.* Down-expression of *MYC* occurred after si[*IGLC*_CR_] transfection. The cells were harvested at 24 h after transfection. Lanes 1 and 2: WILL1, lanes 3 and 4: WILL3, lanes 4 and 5: K562. Odd and even numbers represent si[*Cont*] and si[*IGLC*_CR_]. EtBr staining gel served as a loading control. *D.* Time course of *IGLL5* and *MYC* expression after si[*IGLC*_CR_] transfection. The WILL3 was cultured for 14 h and 24 h after transfection. The RNA blot was concurrently hybridized with the Cλ and MYC probes. The signals derived from the Cλ probe represent *IGLL5* mRNA. Lane 1: 24 h after si[*Cont*]; lane 2: 14 h after si[*IGLC*_CR_], lane 3: 24 h after si[*IGLC*_CR_]. EtBr staining gel served as a loading control.

In our pilot study, we observed that knockdown using si[*IGLC*_CR_] resulted in inhibition of proliferation in IGλ negative cell lines. We then asked whether apoptosis occurred after si[*IGLC*_CR_] treatment. Dead cells significantly increased after si[*IGLC*_CR_] treatment compared with control siRNA in WILL3 (Figs. 3A and 3B). Although statistical significance was not confirmed, WILL1 showed increase of dead cells after si[*IGLC*_CR_] treatment in each paired experiment (Figure S3).

Given that both cell lines have chromosome translocations involving the *MYC* locus and show deregulated expression of *MYC*, we focused on *MYC* expression after si[*IGLC*_CR_] treatment. Both cells showed decreased *MYC* expression 24 h after transfection (Fig. 3C). *MYC* down-expression appeared late after *IGLL5* down-expression (Fig. 3D).

## 4 Discussion

In this study, we detected *IGLL5* expression in two mature B-cell lines lacking IGλ protein. We then found that the expression is controlled by SE as previously predicted. Finally, *IGLL5* down-regulation resulted in cell death and decreased expression of *MYC*.

Five *IGLL* genes have been recognized by the Human Gene Nomenclature Committee [13]. Among the five genes, *IGLL1* and *IGLL5* encode immunoglobulin superfamily proteins, and the remaining are pseudogenes. IGLL1 functions as a surrogate IG molecule expressed in an early differential stage of B-cells [14]. *IGLL5* mRNA expression has been reported in mature B-cells exhibiting IGκ protein [5]. We also detected *IGLL5* mRNA in two mature B-cell lymphoma cell lines, which lacked IGλ protein. Most of the immunoglobulin superfamily proteins function in immunity, cell adhesion or signal transduction [7], but the biological characteristics of the IGLL5 have not been clarified.

Previous ChIP-Seq analyses predicted that SE exists near *IGLL5* in diffuse large B-cell lymphoma and multiple myeloma [5,6]. We found that *IGLL5* mRNA was down-regulated by JQ1, which indicates that *IGLL5* is controlled by SE. The *Cλ* transcripts encoding IGλ protein seemed to not be affected by JQ1 (see FL216 and RPMI8226 in Fig. 2A). The protein coding *IGλ* allele undergoes *Vλ-Jλ-Cλ* recombination which results in the deletion of intervening sequence. The *IGLL5* locates between *Vλ* and *Jλ* regions; thus, the SE of *IGLL5* might be deleted during *Vλ-Jλ-Cλ* recombination in the IGλ protein producing cells. Although the si[*IGLC_CR_*] was reported to target all *Cλ* segments [12], the decreased level of *Cλ* mRNA differed in each cells (Fig. 2C). Different efficacy of *Cλ* down-regulation was observed in a previous study using myeloma cell lines [12], so the difference might be owing to the complexity of the transcriptional regulation of the *IGλ*.

Zhou et al. reported that knockdown of *Cλ* causes apoptosis in IGλ producing myeloma cells resulting from endoplasmic reticulum (ER) stress due to accumulation of uncoupled IG heavy chain protein in the ER [12]. In this study, we used the same siRNA targeting *Cλ*, and observed apoptosis after transfection. The subject cells in our experiments lack IGλ proteins. Therefore, the apoptosis seen in this study should not depend on ER stress. We noted the decreased expression of *MYC* after transfection of si[*IGLC*_CR_]. MYC contributes to tumorigenesis of various malignancies and its expression is regulated by diverse mechanisms. We observed that *MYC* expression was decreased late after *IGLL5* down- expression (Fig. 3D). This time lag might indicate that *IGLL5* down-regulation affects *MYC* expression via intervening processes. Comprehensive gene expression profiles might explore the detailed link between *IGLL5* and *MYC* expression.

We observed apoptosis after si[*IGLC*_CR_] transfection to *IGLL5* expressing mature-B cell lines. However, WILL1 did not show a significant increase in dead cells. Moreover, although WILL3 showed a statistically significant increase in cell death after siRNA treatment, the increase ratio was less than 10 %. The siRNAs may therefore have partial effects on *IGLL5* mRNA expression. One of the reasons for the partial effects is presumably due to transient efficacy, as suggested in Fig. 3D. To obtain sufficient efficacy, other efficient gene silencing methods, such as lentiviral vector or gene editing methods, should be used in future experiments.

In conclusion, our results suggest that *IGLL5*, an immunoglobulin superfamily protein encoding gene, is controlled by super-enhancer, and might contribute to cell survival affecting *MYC* expression. Further study with larger sample sizes should need to explore biological and clinical significance of *IGLL5* in B-cell malignancies.

## CRediT authorship contribution statement

**Hiroki Hosoi:** Data curation, Investigation, Writing – original draft. **Shotaro Tabata:** Investigation. **Hideki Kosako:** Investigation. **Yoshikazu Hori:** Investigation. **Tadashi Okamura:** Investigation. **Yusuke Yamashita:** Investigation. **Kota Fujimoto:** Investigation. **Daiki Kajioka:** Investigation. **Kentaro Suzuki:** Investigation, Supervision. **Motomi Osato:** Project administration, Supervision, Writing – original draft. **Gen Yamada:** Project administration, Supervision, Writing – original draft. **Takashi Sonoki:** Data curation, Funding acquisition, Investigation, Methodology, Project administration, Supervision, Validation, Writing – original draft, Writing – review & editing.

## Declaration of competing interest

None.

## Appendix Supplementary materials

Image, application 1
