# Supplementary material for: IGLL5 controlled by super-enhancer affects cell survival and MYC expression in mature B-cell lymphoma
Source: Leuk Res Rep. 2024 Feb 22;21:100451. doi: 10.1016/j.lrr.2024.100451 (PMC10912717; doi:10.1016/j.lrr.2024.100451)
Supplement: Supplementary file 1 [file mmc1.docx]

**Supplementary table**

| Cell line | Immunoglobulin (Ig)  protein | Chromosome translocation involving  Ig genes | Disease |
| --- | --- | --- | --- |
| MD901 | surface IgM/IgD/Igκ | t(3;22)(q27;q11)  t(8;22)(q24;q11) | DLBCL* |
| FL218 | surface IgG/Igλ | t(14;18)(q32;q21) | FL^#^ |
| WILL1 | Igκ secrete | t(6;8;14)(q27;q24;q32) | DLBCL* |
| WILL3 | None | t(14;18)(q32;q21)  t(8;22)(q24;q11) | DLBCL* |

MD901^1)^ and FL218^2)^ were kindly provided from Dr. T Miki at Tokyo Medical and Dental　University and Dr. M Nishikori at Kyoto University, respectively. WILL1^3)^ and WILL3^4)^ were established in our laboratory. *DLBCL: diffuse large B-cell lymphoma, ^#^FL: follicular lymphoma.

**Supplementary references**

1) Miki T, Kawamata N, Arai A, Ohashi K, Nakamura Y, Kato A, Hirosawa S, Aoki N. Molecular cloning of the breakpoint for 3q27 translocation in B-cell lymphomas and leukemias. Blood 1994; 83: 217– 22.

2) Amakawa R, Fukuhara S, Ohno H, Tanabe S, Horii M, Matsuyama F, Kato I, Kakita T, Nagauchi O. Amplified and rearranged bcl-2 gene in two lymphoma cell lines, FL-218 and FL-318, carrying a 14;18 translocation. Cancer Res. 1990; 50: 2423-8.

3) Uneda S, Gotoh M, Sonoki T, Nishida K, Nakamura Y, Kurimoto M, Hanaoka N, Matsuoka H, Taniwaki M, Nakakuma H. Establishment of CD5 and CD10 double-positive mature B-cell line, WILL1, showing complex 8q24 translocation involving 14q32 and 6q27. Int J Hematol. 2008 ;88 :536-542.

4) The WILL3 was established from the bone marrow cells of a 60-years-old Japanese woman who presented multiple lymph-nodes swelling. The pathological diagnosis of the lymph-node was DLBCL. The tumor cells lacked IGκ and IGλ. Chromosome analysis revealed multiple abnormalities including t(14;18)(q32; q21) and t(8;22)(q24;q11). We cloned the t(14;18)(q32;q21) and the t(8;22)(q24;q11) by long-distant inverse PCR. The *BCL2* and *MYC* breakpoints fell to the ICR region of *BCL2* and ~5 kb downstream of *MYC*, respectively.

1, Phenotypic and karyotypic analysis of WILL3


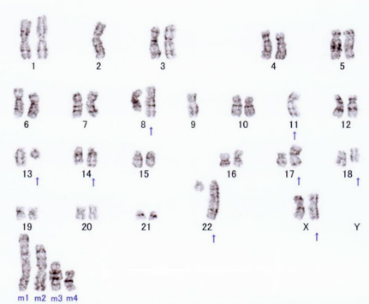

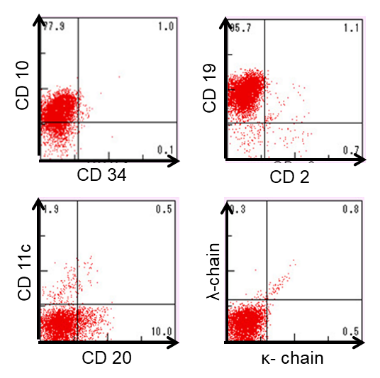


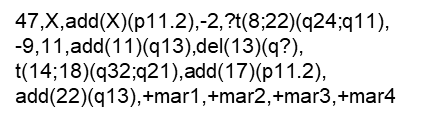


2. Southern blot and LDI-PCR analyses of WILL3

**
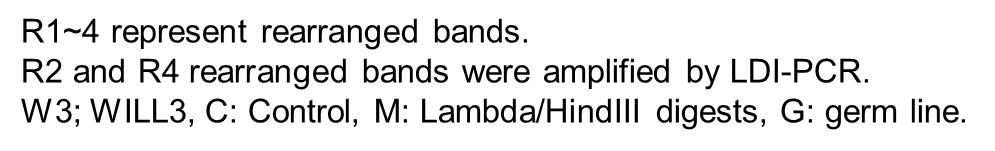

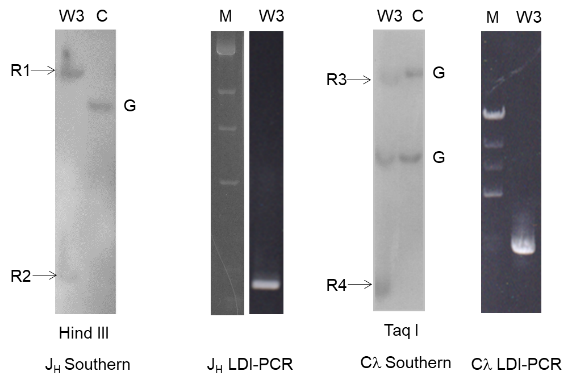
**3. Junctional sequence of t(14;18)(q32;q21) (Bald, small, plain letters represent 18q21, inserted and 14q32 sequences, respectively)

**TTCTACAATTTGAGCCTCAATTAATGTTTGTTTACTTATCTGCTAATCTCACTATTTTTTCTAAATTCGTTCTCAGTAAGTGAGAGTGCAG**gaaaacatgtatTACTACTACTACTACGGTATGGACGTCTGGGGCCAAGGGACCACGGTCACCGTCTCCTCAGGTAAGAATGGCCACTCTAGGGCCTTTGTTTTCTGCTA

4. Junctional sequence of t(8;22)(q24;q11) (Bald, small, plain letters represent 8q24, inserted and 22q11 sequences, respectively)

**AGCTTGGATGAAGCATGTAACCTATGTCAGGGAGGAAGCCCTGGTGTGTCAAAGGCAGCAAGA***aac*TCGGCGGAGGGACCAAGCTGACCGTCCTAGGTGAGTCTCTTCTCCCCTCTCCTTCCCCACTCTTGGGA

**Supplemental figures**

**Figure S1**.


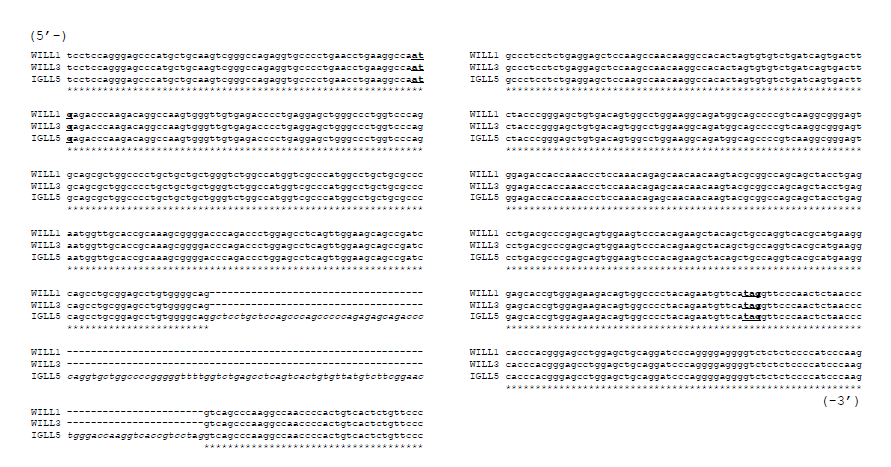


Comparison of nucleotide alignments of RT-PCR products derived from WILL1 and WILL3 with *IGLL5* mRNA. Initiation (atg) codon and stop (tag) codon of *IGLL5* isoform 2 are shown in underlined bald letters. Italic letters indicate the exon 2 (*Jλ1* segment) that is deleted in WILL1 and WILL3. WILL1 and WILL3 express *IGLL5* transcription isoform 2 that encode 139 amino acids protein. We used MAFFT version 7 (https://mafft.cbrc.jp/alignment/server/) to compare the three sequences. The nucleotide alignments are shown from upper left (5’-) to lower right (-3’).

**Figure S2.**


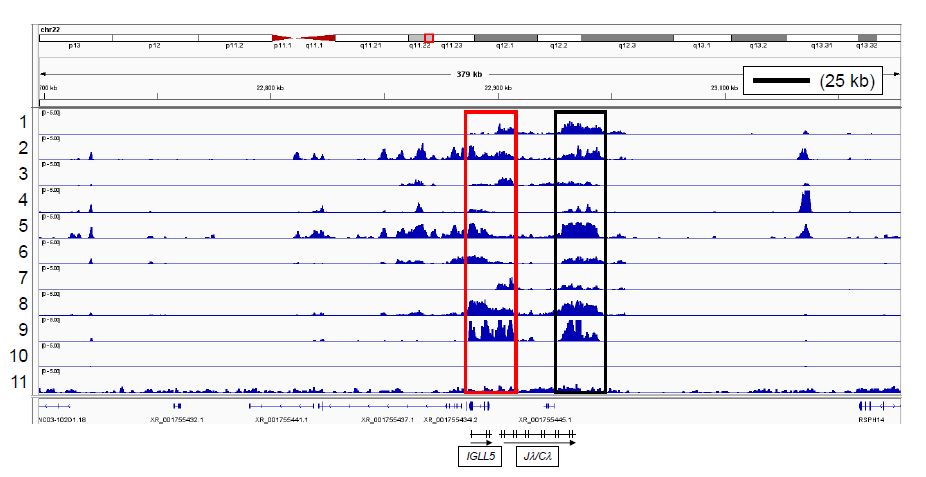


The ChIP-seq data of various mature B-cell lymphoma cell lines are shown. Peak call represents sequence accumulation using anti-H3K27ac antibody. The published data at ChIP-Atlas (https://chip-atlas.org/) are visualized by IGV_2.8.9 software. Peaks in red box represent large active enhancer near *IGLL5* region. Peaks in black box are considered to represent 3’ lambda enhancer^1), 2)^. Lane 1: MD901 (IgM/D/κ), lane 2: Karpas-422 (IgM/κ), lane 3: JEKO1 (IgM/κ), lane 4: OCY-LY-7 (IgM/κ), lane 5: SU-DHL-4 (IgG/κ), lane 6: SU-DHL-6 (IgM/κ), lane 7: GRANTA-519 (IgM/λ), lane 8: OCY-LY-3 (IgG/λ), lane 9: Raji (IgM). Surface IG protein is shown in parenthesis. Phenotypic characteristics of the cell lines were obtained from German Collection of Microorganisms and Cell Cultures GmbH (DSMZ) (https://www.dsmz.de/). Lanes 10 and 11 are Jurkat (T-ALL cell line) and NB-4 (AML cell line), respectively. Jurkat and NB-4 represented negative controls lacking high peak near *IGLL5*. The *IGLL5* and *Jλ* /*Cλ* complex’s loci are drawn in red and black, respectively, under peak call figure. There are seven Jλ/Cλ segments. Note *IGLL5* includes Jλ1 and Cλ1 as the second and third exons. Arow indicates transcriptional orientation. The detailed ChiP-Seq methods for each cell line are available at corresponding web site as described follows. MD901: https://chip-atlas.org/view?id=SRX1665943, Karpas-422: https://chip-atlas.org/view?id=SRX6430561, JEKO-1: https://chip-atlas.org/view?id=SRX1048464, OCI-LY-7: https://chip-atlas.org/view?id=SRX1048479, SU-DHL-4: https://chip-atlas.org/view?id=SRX5985311, SU-DHL-6: https://chip-atlas.org/view?id=SRX683856 GRANTA-519: https://chip-atlas.org/view?id=SRX1048462, OCI-LY-3: https://chip-atlas.org/view?id=SRX16791294, Raji: https://chip-atlas.org/view?id=SRX498151, Jurkat (T-ALL cell line): https://chip-atlas.org/view?id=SRX398094, NB-4 (AML cell line) : https://chip-atlas.org/view?id=SRX13130617. All ChIP-Seq data and methodologies are available at the above web sites on 02/January/2024.

References

1) Asenbauer H, Klobeck HG. Tissue-specific deoxyribonuclease I-hypersensitive sites in the vicinity of the immunoglobulin C lambda cluster of man. Eur J Immunol. 1996 Jan;26(1):142-50. doi: 10.1002/eji.1830260122.

2) Gabriele Combriato, H.-Gustav Klobeck; Regulation of Human Igλ Light Chain Gene Expression by NF-κB. J Immunol 1 February 2002; 168 (3): 1259–1266. https://doi.org/10.4049/jimmunol.168.3.1259

**Figure S3.**


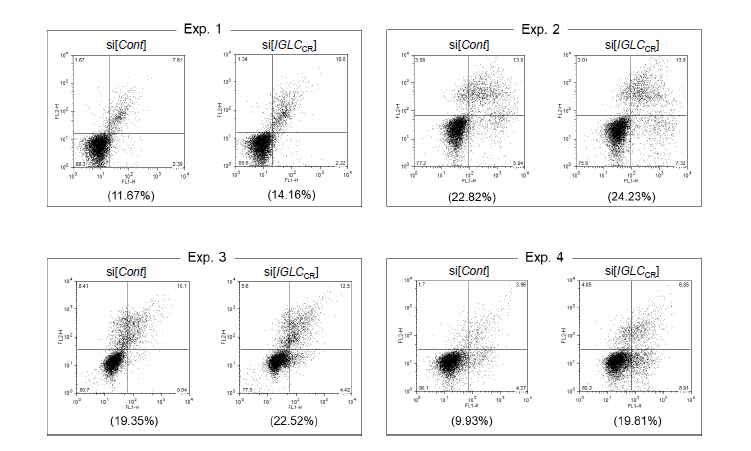


Apoptosis assay of WILL1 24h after si[*IGLC_CR_*] transfection. Four independent paired experiments were shown. Numbers in parenthesis represent dead cells (%).
